# A hydrofluoric acid-free method to dissolve and quantify silica nanoparticles in aqueous and solid matrices

**DOI:** 10.1038/s41598-019-44128-z

**Published:** 2019-05-28

**Authors:** David Bossert, Dominic A. Urban, Mattia Maceroni, Liliane Ackermann-Hirschi, Laetitia Haeni, Phattadon Yajan, Miguel Spuch-Calvar, Barbara Rothen-Rutishauser, Laura Rodriguez-Lorenzo, Alke Petri-Fink, Fabienne Schwab

**Affiliations:** 1 Adolphe Merkle Institute, University of Fribourg, Chemin des Verdiers 4, 1700 Fribourg, Switzerland; 2 Chemistry Department, University of Fribourg, Chemin du Musée 9, 1700 Fribourg, Switzerland; 3 Nano4Enviroment Unit, Water Quality Group, INL - International Iberian Nanotechnology Laboratory, 4715-330 Braga, Portugal

**Keywords:** Environmental sciences, Environmental chemistry, Analytical chemistry, Materials science, Nanoscience and technology

## Abstract

As the commercial use of synthetic amorphous silica nanomaterials (SiO_2_-NPs) increases, their effects on the environment and human health have still not been explored in detail. An often-insurmountable obstacle for SiO_2_-NP fate and hazard research is the challenging analytics of solid particulate silica species, which involves toxic and corrosive hydrofluoric acid (HF). We therefore developed and validated a set of simple hydrofluoric acid-free sample preparation methods for the quantification of amorphous SiO_2_ micro- and nanoparticles. To circumvent HF, we dissolved the SiO_2_-NPs by base-catalyzed hydrolysis at room temperature or under microwave irradiation using potassium hydroxide, replacing the stabilizing fluoride ions with OH^−^, and exploiting the stability of the orthosilicic acid monomer under a strongly basic pH. Inductively coupled plasma – optical emission spectroscopy (ICP-OES) or a colorimetric assay served to quantify silicon. The lowest KOH: SiO_2_ molar ratio to effectively dissolve and quantify SiO_2_-NPs was 1.2 for colloidal Stöber SiO_2_-NPs at a pH >12. Fumed SiO_2_-NPs (Aerosil^®^) or food grade SiO_2_ (E551) containing SiO_2_-NPs were degradable at higher KOH: SiO_2_ ratios >8000. Thus, hydrofluoric acid-free SiO_2_-NP digestion protocols based on KOH present an effective (recoveries of >84%), less hazardous, and easy to implement alternative to current methods.

## Introduction

Knowing the concentrations of amorphous silica particles is essential in numerous areas of science and technology. Several million tons of this material are produced each year^1^. However, the analysis of particulate silica species (here termed SiO_2_) is challenging. A major reason for the lack of knowledge in biomedical sciences on SiO_2_ nanoparticle (SiO_2_-NP) cell interactions or long-term fate and effects in the body^2^ is the costly, time-consuming, and hazardous method required for the analysis of Si. As a result of the challenging analytics involved with the quantification of SiO_2_-NPs, most environmental and toxicological studies are performed with nanoparticles that are less environmentally relevant, but easier to analyze (*e*.*g*., gold^3^ or quantum dots). Other fields relying on silicon analytics including micro- and nano-sized SiO_2_-NPs are fate research on SiO_2_ nano-agrochemicals, micronutrient cycling of orthosilicic acid (Si(OH)_4_) in agriculture^4,5^, and the biogeochemical impacts of phytoplankton such as diatoms^6,7^. In daily life, food grade SiO_2_-NPs are present as a part of food grade SiO_2_ (E551) that is used in various products (*e*.*g*. in table salt as an anticoagulant). Their expected daily intake is 1.8 mg kg^−1^ ^8^. Due to the release of studies that indicate this nanomaterial may cause a pro-inflammatory response^9–11^, manufacturers and consumers of products containing E551 have a direct interest in knowing their SiO_2_ content. A promising emerging approach that directly detects intact, *i*.*e*. not previously digested, SiO_2_-NPs is single-particle mass spectrometry^12^. For now, this method is limited to a specific particle size range and requires highly specialized laboratory equipment and personnel. The need for simpler, inexpensive, and broadly applicable Si quantification methods to quantify SiO_2_-NP concentrations cannot be overstated.

To date, the analytical methods of choice for Si quantification are the detection of Si(OH)_4_ using Si-molybdate complexes by UV-Vis^13,14^ (colorimetry) or detection of Si by inductively coupled plasma – optical emission spectroscopy (ICP-OES). All the modern colorimetric assays to quantify Si refer back to the fundamental work on silicomolybdic acid complexes achieved in the 1950s^13,15–17^, which has been continuously refined until now^6,18,19^. Due to the advent of complementary methods, certain limitations of colorimetry have been discovered^20^. While providing excellent detection limits in simple matrices in the low µmol L^−1^ or even nmol L^−1^ range^6,21^, the detection of the colored silicomolybdic acid complexes^15–17,22^ can suffer from interferences with a broad palette of contaminants such as iron, phosphates, nitrate, ammonia, or sugars, making the quantification of Si in this manner challenging in complex mixtures^20^. For such matrices, the more recent and robust approaches used to detect Si are inductively coupled plasma – optical emission spectrometry (ICP-OES) or - mass spectrometry (MS). Similar to colorimetry, the detection *via* ICP-OES requires the complete solubilization of the sample to ensure accurate results and instrument stability. Microwave-assisted closed-vessel digestion is widely used for sample solubilization and offers several advantages such as high reaction pressures/temperatures, high throughput, and low risk of contamination^23^. The primary reagents used to break down the organic matter are mixtures of strong concentrated acids and/or oxidative reagents, *e*.*g*. HNO_3_, HCl, H_2_SO_4_, and H_2_O_2_. Both colorimetric and ICP-OES sample preparation protocols have in common that concentrated hydrofluoric acid is used as a standard ingredient to break down the poorly soluble particular SiO_2_ fraction and to stabilize Si in solution in soluble Si-F complexes. Hydrofluoric acid is highly hazardous, especially once it is in contact with skin, and therefore requires expensive and time-consuming safety precautions. Additional neutralization steps before injection into the instrument are needed because free hydrogen fluoride (HF) is highly corrosive for tubings and glass parts^24,25^. Silicon quantification methods under acidic conditions are therefore either prone to false-positive results, and/or include hazardous chemicals, are time-consuming, and are expensive.

Since SiO_2_ dissolution or extraction is an essential step in Si quantification, alternative hydrofluoric acid-free solubilization and extraction protocols for silica have been explored. Digestion protocols under basic conditions use for example lithium metaborate (LiBO_2_), lithium tetraborate (Li_2_B_4_O_7_), tetramethylammonium hydroxide (TMAH), and alkali bases^26,27^. Fusion techniques involving LiBO_2_ and Li_2_B_4_O_7_ are often used in geology to analyze soils and rocks with high Si contents, where large sample masses are available. However, for the analysis of samples containing Si in the lower mg range, these techniques have the drawback of being an open vessel digestion of only solid samples with high temperatures, low throughput, and the introduction of a high concentration of salt into the sample, requiring high dilution, and increasing the limits of quantification^27^. Similar to HF, TMAH is highly poisonous^28^. Concentrated NaOH and KOH can break down crystalline (*e*.*g*. quartz) samples and are less hazardous alternatives to extract Si from *e*.*g*. quartz, feldspar, and kaolin^29^. However, for samples containing organic carbon these bases have to be combined with strong oxidizers, which can have detrimental effects on the PTFE (Teflon) digestion vessels and the ICP-OES torch (*e*.*g*. NaOH and H_2_O_2_)^30^. Initial attempts to dissolve and quantify low amorphous SiO_2_ concentrations in plant samples used H_2_O_2_ in conjunction with KOH^30^, or low KOH: SiO_2_ ratios of 1.2 to analyze silicate species in amorphous silica gel^18^. Despite these first successes using KOH, the state of the art remains to dissolve SiO_2_ in acidic protocols using hydrofluoric acid, largely due to a lack of validation and knowledge on the application scope of this non-traditional approach.

**Figure 1:**
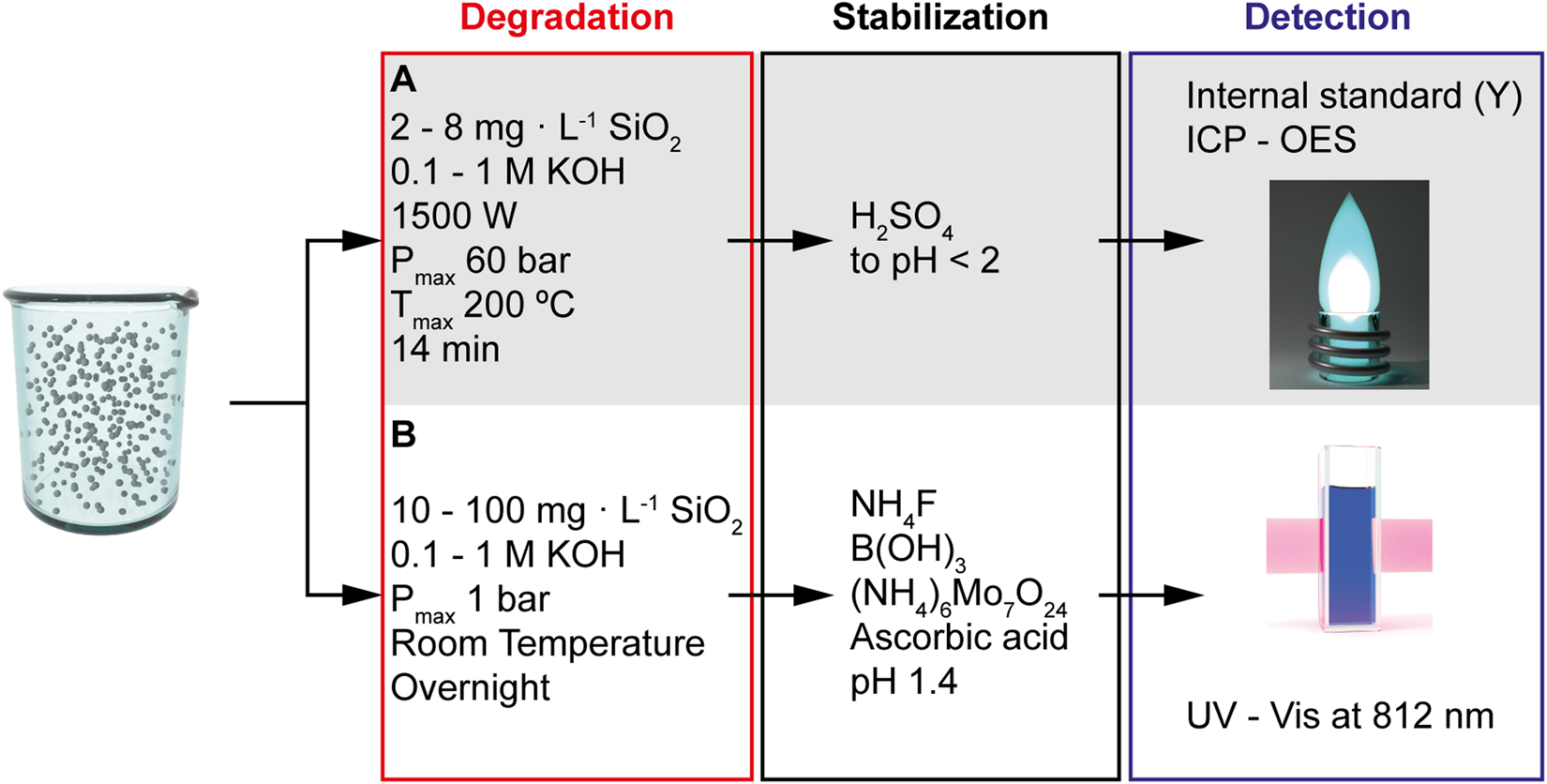
Key steps and reagents used to hydrolytically degrade SiO_2_ nanoparticles under basic conditions using potassium hydroxide, and detect dissolved Si and Si(OH)_4_, respectively, under acidic conditions. The SiO_2_ concentrations stated are those used for the method development. (**A**) HF-free procedure for detection by inductively coupled plasma – optical emission spectrometry (ICP-OES) method suitable for complex matrices and accurate detection of low Si concentrations. (**B**) Procedure for detection by the colorimetric method using a UV-vis spectrophotometer. This method involves *in situ* HF, was used to validate method A, and is suitable for simple sample matrices.

A validated SiO_2_ solubilization method based on KOH can significantly reduce the hazards and time to quantify Si. A hydrofluoric acid-free approach is more ecologic and economic due to the reduced use of disposable personal protection equipment, less toxic waste, and less corrosion damage to the equipment. The aims of the present work were therefore to (a) explore and validate the potential of KOH to digest amorphous particulate SiO_2_ for the quantification of Si by direct comparison with an approach that involves *in situ* hydrofluoric acid (Fig. 1); and (b) use the resulting KOH digestion methods to quantify the concentration and recovery of three different types of SiO_2_ (colloidal, fumed, and food grade) in matrices of increasing complexity, ranging from water to cell culture medium and food.

## Results and Discussion

### Molar ratio of KOH: SiO_2_ for complete SiO_2_ dissolution—mechanism

**Figure 2:**
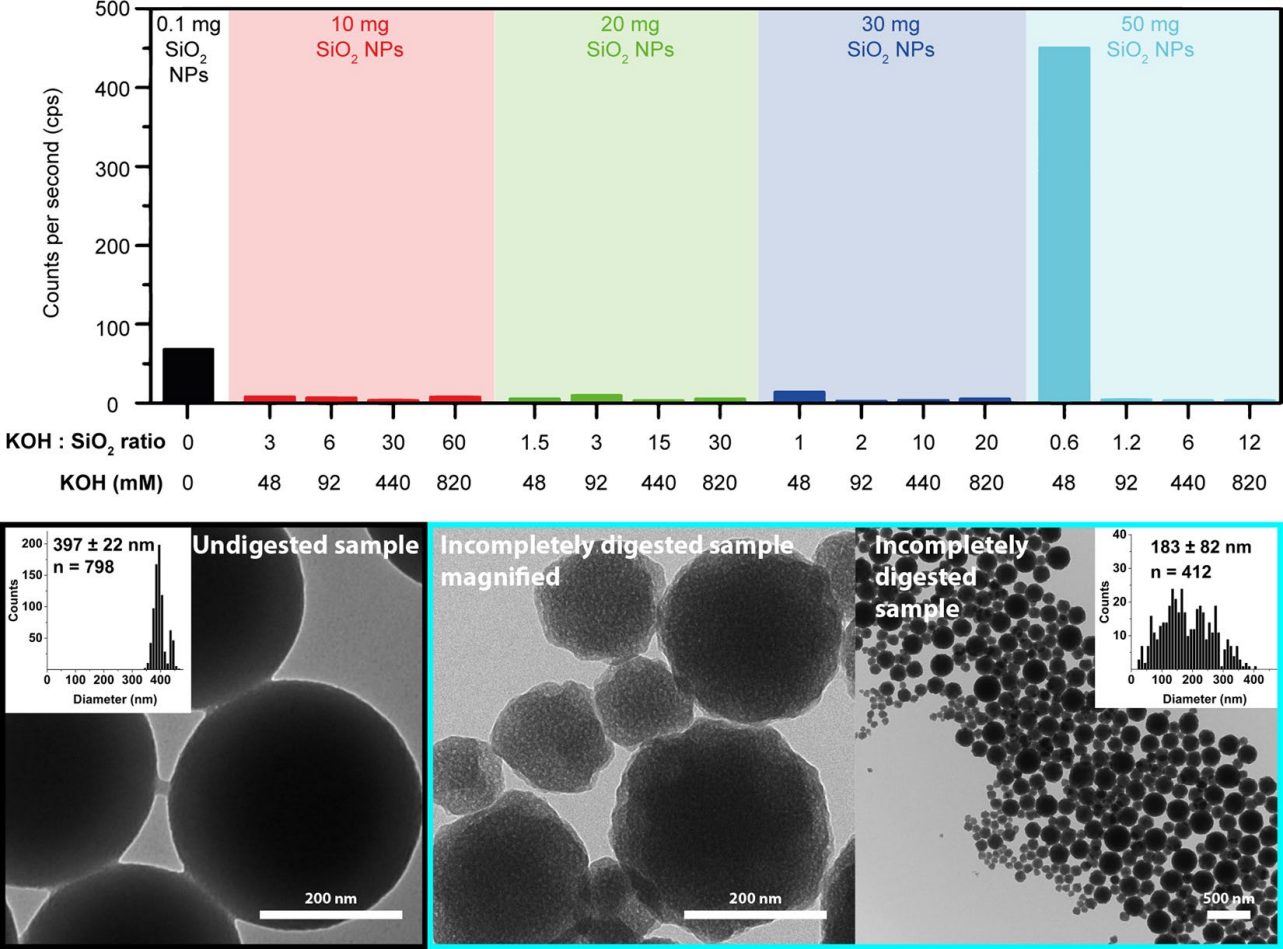
Results of digestion methods tested to find the optimal KOH: SiO_2_ ratio that allowed for a complete dissolution of SiO_2_-NPs. Dynamic light scattering (DLS) particle count rates and transmission electron microscopy (TEM) images are shown. High count rates represent undigested or partially digested colloidal SiO_2_-NPs. The lowest possible potassium hydroxide concentration for complete solubilization of the SiO_2_-NPs (digestion method: High_SiO_2_) was 0.092 M. In these or higher tested KOH concentrations (molar ratio of 1.2–60.1 KOH: SiO_2_), all SiO_2_-NPs dissolved. The high particle count rate in the 48 mM KOH sample with a molar KOH: SiO_2_ ratio of 0.6 shows that the lower KOH concentration did not fully dissolve the 50 mg of SiO_2_-NPs. For these incompletely digested SiO_2_-NP samples, the TEM micrographs revealed a broadening of the size, an increase in surface roughness, and the presence of nanopores compared to the original SiO_2_-NPs.

The results of the method optimization using the High_SiO_2_ digestion method outlined in the method section and Table 1 are presented in Fig. 2. The dissolution of colloidal SiO_2_ into Si(OH)_4_ species was less dependent on the concentration of KOH, and more on the ratio of KOH to SiO_2_, which optimally is >1.2, and the pH, which should be >12. A volume of 10.0 mL of 0.1 M KOH (final concentration 82 mM) solubilized up to 50 mg of colloidal SiO_2_-NPs in suspension (Fig. 2). This corresponds to a molar ratio of 1.2 KOH: SiO_2_, in line with the results from Yang *et al*.^19^. The same volume of 0.05 M KOH still dissolved up to 30 mg colloidal SiO_2_-NPs (molar ratio: 1.0 KOH: SiO_2_), but did not dissolve 50 mg SiO_2_-NPs anymore (molar ratio: 0.6 KOH: SiO_2_), apparent from the high particle counts per second detected by DLS in that particular sample (Fig. 2). A slightly elevated DLS signal was also observed for the molar ratio of 1.0 KOH: SiO_2_. These results demonstrate that at least an equimolar concentration of KOH and optimally an excess of >20% is needed to dissolve SiO_2_. The constant ratio suggests that KOH fulfills a two-fold purpose: (a) installing a pH of >12 for the base-catalyzed hydrolytic degradation of hydrated silica^31^, and (b) neutralizing the Si(OH)_4_ liberated during this reaction to maintain the high pH. The threshold pH of >12 corresponds to the 14 mM KOH that are not neutralized by Si(OH)_4_ in the sample digested with 1.2 KOH: SiO_2_ (82 mM KOH, 68 mM SiO_2_), and is in agreement with the pH of 9–12 reported by Croissant *et al*. to dissolve SiO_2_-NPs^31^. We therefore adapted 0.1 M KOH and 1.2 KOH: SiO_2_ ratio as minimum values for further digestions for colloidal SiO_2_-NPs, and slightly more for fumed SiO_2_ NPs based on our observations (discussion below).

**Table 1:** Investigated digestion methods for samples containing SiO_2_.

Method	Digestion				Analysis		
	max. SiO_2_ (mg L^−1^)	max. KOH (M)	range of molar ratio KOH: SiO_2_	Digested by	max. Si (mg L^−1^)	max. KOH (mM)	Analyzed by
High SiO_2_	4110	0.958	0.6–60.1	Microwave	1921	958	DLS
KOH0.1	8.53	0.06	423–1922		1.98	30	ICP
KOH0.5	70	0.5	0.4–1.7		0.75	100	
KOH1.0	4.50	0.60	8017–36916		1.05	300	
RT + KOH0.1	100	0.1	60.1	RT	4.45	10	UV-Vis
RT + KOH1.0	2.61	0.6	13803–14704		0.57	300	ICP
	85	1	462–707		2.39	39	UV-Vis

High_SiO_2_: high SiO_2_-NP concentrations were used to find the lowest KOH concentration leading to complete solubilization. KOH0.1, KOH0.5, KOH1.0: different KOH concentrations were tested to assess Si recovery in simple and complex matrices. RT + KOH0.1, RT + KOH1.0: the solubilization of SiO_2_-NPs at room temperature (RT) was tested (recoveries: refer to Table 2).

For further investigation on the dissolution mechanism, the incompletely digested sample containing 50 mg colloidal SiO_2_-NPs in suspension with the insufficient 0.6 KOH: SiO_2_ ratio (Fig. 2) was dialyzed after the digestion against water for 1 d to remove KOH and dissolved Si species, and then inspected by transmission electron microscopy (TEM, Fig. 2). The structure of these partially dissolved SiO_2_-NPs revealed electron-transparent nanopores and more surface roughness compared to the dense, non-porous, and smooth structure of freshly synthesized SiO_2_-NPs (Fig. 2), confirming results of Li *et al*., who, based gas adsorption analysis results, ascribed some nanoporosity to colloidal SiO_2_-NPs due to aggregation-based NP growth^32^. An alternative explanation for the porosity is that the dissolution does not progress from the surface towards the core, but is targeted on specific silanol bonds^31^ throughout the molecular structure. These observations are in line with Park *et al*., who showed that SiO_2_-NPs undergo a shape evolution due to Si-O bond-breaking and bond-making caused by hydroxyl ions, leading to rearrangement of high-energy bonds in the core^33,34^. No remaining NPs were observable by TEM in the samples digested using ratios >0.6 KOH: SiO_2_ (Fig. 2).

### Method development

#### Comparison of calibration types

**Figure 3:**
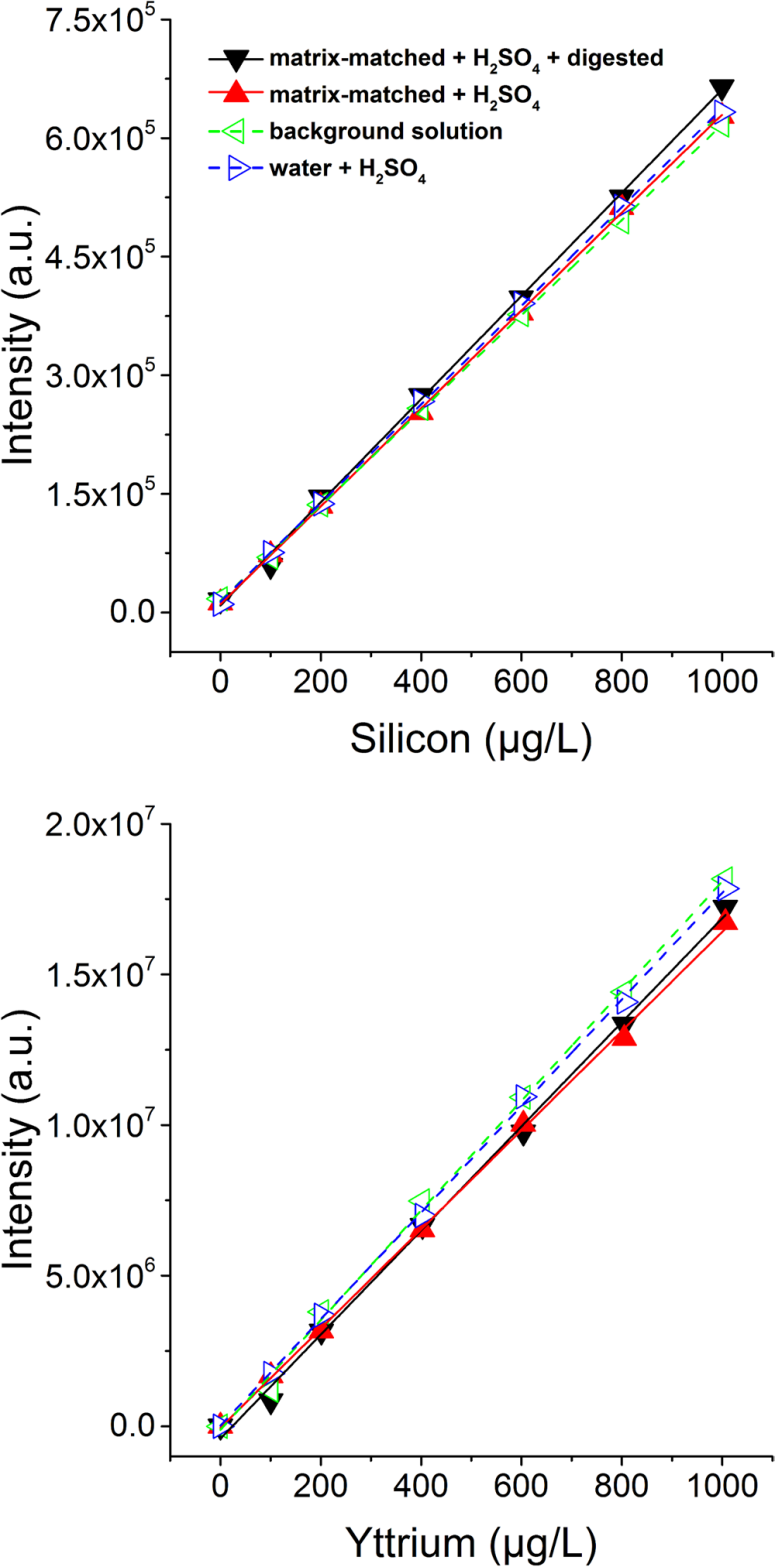
Four calibrations of Si in matrices of increasing complexity are compared. Water + H_2_SO_4_: Si in water, acidified by H_2_SO_4_. Background solution: Si in aqueous acidic background solution (2% HNO_3_, 0.5% HCl). Matrix-matched + H_2_SO_4_: Si with added KOH (3 mL, 0.1 M), without microwave digestion, acidified by H_2_SO_4_. Matrix-matched + H_2_SO_4_ + digested: Si with added KOH (3 mL, 0.1 M), with microwave digestion, acidified by H_2_SO_4_.

**Figure 4:**
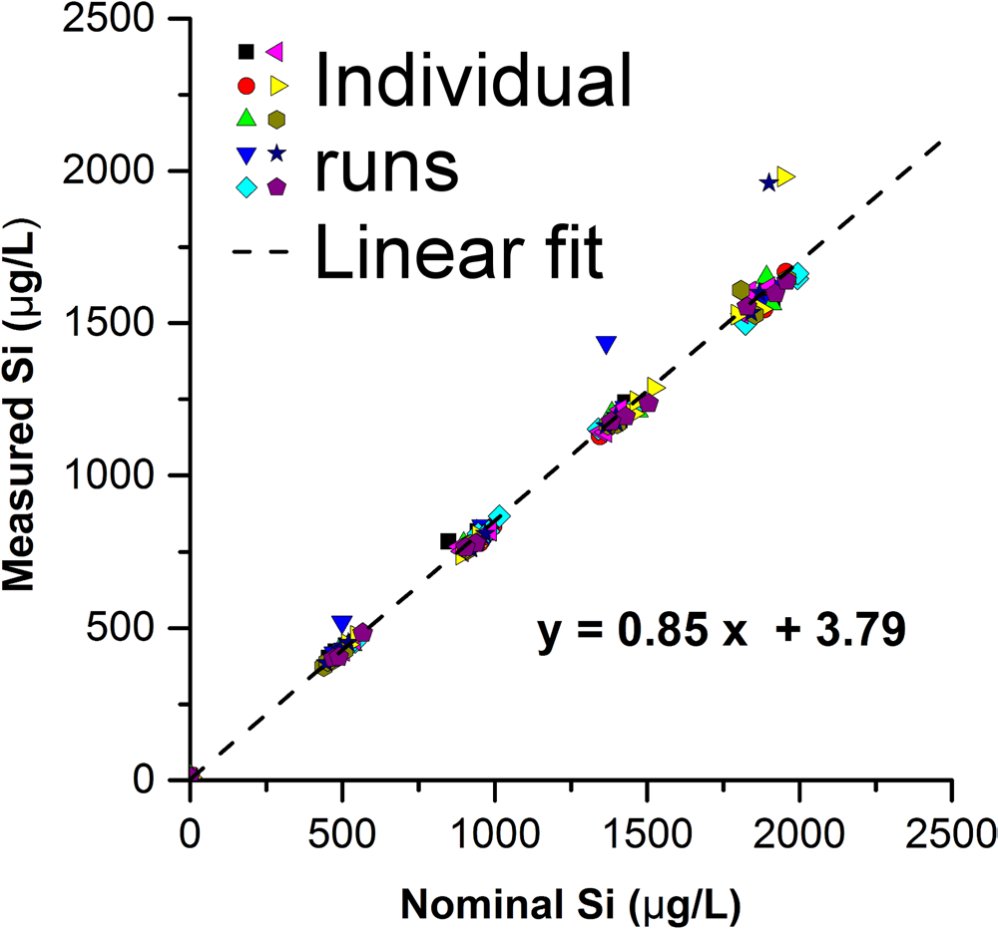
Recovery and repeatability for the inductively coupled plasma – optical emission spectrometry (ICP-OES) measurements of SiO_2_-NPs. One hundred twenty samples and ten blanks were measured to test the performance of the Si quantification (digestion method KOH0.1). Gravimetrically calculated nominal Si concentrations *versus* the measured internal yttrium standard corrected Si concentrations are shown. The dashed line is the linear fit through all measurements. The method was repeatable and resulted in a recovery of 85.3 ± 2.2% in the concentration range of 0–2 mg Si L^−1^ of silicon dioxide.

Four calibrations with increasing degrees of complexity (water + H_2_SO_4_; acidic background solution (BgS, 2% HNO_3_, 0.5% HCl); matrix-matched + H_2_SO_4_; and matrix-matched + H_2_SO_4_ + digested) are shown in Fig. 3 and their fitting parameters in Supplementary Table S2. As apparent from the high *R*^*2*^ (0.9987–0.9998), both Si and Y were stable in the concentration range of the calibrations under all conditions. As expected, calibrations exclusively containing acids showed the most stable Si signal (*R*^*2*^ = 0.9998), and the most complex calibration was slightly more unstable (*R*^*2*^ = 0.9987). A similar trend was observed for the signal of the internal standard yttrium. Only subtle signal suppression of Si or Y due to the matrix were observable: the maximal relative difference between the sensitivity of the different calibrations was 7.7% for Si and 9.5% for Y. For Si, the highest sensitivity (652 ± 9.5 counts/(µg L^−1^)) was observed for the matrix-matched + H_2_SO_4_ + digested calibration, and the lowest for the BgS calibration (602 ± 3 counts/(µg L^−1^)). The digestion and addition of 0.1 M KOH moderately stabilized free Si. However, Fig. 3 shows that neither the acids used (2.25 M H_2_SO_4_, 0.5% HCl, and 2.0% HNO_3_), nor the 0.1 M KOH, nor the digestion in the microwave led to a change of the Si signal noticeable in the statistical scatter of the data. For Y, the trends in matrix effects were somewhat different than for Si, and the highest sensitivity (18204 ± 321 counts/(µg L^−1^)) was observed for the BgS calibration, in agreement with HSAB theory (stabilizing effect of soft nitrate ligands on the soft Y metal ions which is less effective for hard Si ions)^35^. The variability of the Y intercept was somewhat increased due to an accidental systematic second addition of internal standard which had to be corrected in the data by subtraction. The Y calibrations suffered from slight sensitivity loss under the matrix-matched KOH conditions by ~2–7%. Nevertheless, the absolute sensitivity for Y was excellent throughout all experiments. We therefore used the matrix-matched + H_2_SO_4_ + digested calibrations with the highest sensitivity for Si for all measurements shown in Fig. 4 and Table 2.

**Table 2:** Recoveries and detection limits for amorphous SiO_2_-NPs.

Detection method	Digestion method	SiO_2_ source	Matrix	Recovery ± s.d. (%)	BEC	LOQ	LOD
					(µg SiO_2_ L^−1^)		
ICP-OES	KOH0.1	colloidal	water	85 ± 2	24.5	80.1	41.1
	KOH0.5		DMEM	84 ± 20	48.2	216.3	98.6
	KOH1.0	fumed	water	114 ± 25	63.7	67.6	64.8
		colloidal	tomato sauce^a^	124 ± 5	n.d.	n.d.	n.d.
		food grade	seasoning^a^	95 ± 13	n.d.	n.d.	n.d.
	RT + KOH1.0	colloidal	water	84 ± 4.9	113	296	168
		fumed		105 ± 1.4	113	296	168
Colorimetry	RT + KOH0.1	colloidal		111 ± 7	115	264	148
		fumed		67 ± 2	22	430	144
	RT + KOH1.0			76 ± 9	120	307	176

Colloidal Stöber SiO_2_-NPs; fumed Aerosil^®^ SiO_2_-NPs; and food grade SiO_2_, also known as E551. The SiO_2_-NPs were analyzed in matrices of increasing complexity: water, cell culture medium (DMEM), potato seasoning, and tomato sauce. The numbers in the digestion method names are the KOH concentrations used in mol L^−1^.

s.d.: standard deviation. n.d.: not determined. BEC: background equivalent concentration. LOD: instrument limit of detection. LOQ: instrument limit of quantification. RT: room temperature. ICP: Inductively coupled plasma – optical emission spectrometry. ^a^The food matrix was pre-digested as mentioned in section *Pre-digestion of samples containing food matrix*. Potato seasoning and tomato sauce recovery relative to mass after pre-digestion of the food matrix.

#### Repeatability and recovery

The measured concentrations of the SiO_2_-NP suspensions (Table 1) digested *via* the method KOH0.1 are compared with their calculated concentrations of Si in Fig. 4. A total recovery of Si/SiO_2_ of 85 ± 2% was achieved with an instrument limit of detection of 41 µg L^−1^ and an instrument limit of quantification of 80 µg L^−1^ SiO_2_ (Table 2). The method was linear in the investigated range of injected Si (373–1981 µg L^−1^) which corresponds to 1.88–8.53 mg L^−1^ SiO_2_ during the digestion. The relative error of the Si sensitivity, which can be attributed to measurements being taken over the course of multiple days by different investigators with different calibration matrices, was 31%. Three outliers are present among the hundred-twenty repeated measurements in Fig. 4. We attribute them to human pipetting errors. In practice, such errors can be detected and eliminated by analyzing, as in the present study, at least *n* = 3 replicate samples. While no outliers were deleted in the present study to present the reader with a realistic dataset, performing for example a Grubbs outlier test can identify such anomalies. In potential future large-scale applications, a robotic pipetting system can prevent such outliers. Overall, the repeatability of the measurements of concentration series prepared individually, digested in different microwave runs, and measured on the same day was very high (Fig. 4). This demonstrates that there is no significant buildup of Si in the instrument within one run, and the selected rinsing time of 55 s (10% HNO_3_) between samples was sufficient. We found, however, that it is necessary to clean the detector window in regular intervals and to thoroughly rinse the instrument with 10% HNO_3_ and Milli-Q (18.2 MΩ · cm) after each run.

We expected the recovery of Si in ICP-OES to be proportional to the stability of free Si(OH)_4_, and inversely proportional to the fraction of re-polymerized Si(OH)_4_ in the sample. The polymerization of silica is accelerated under several conditions such as pH >2, high temperature, and ionic strength >0.2 M^19,36^. Here, the pH was adjusted by the addition of H_2_SO_4_ to a pH of <2 to minimize polymerization and push the equilibrium towards orthosilicic acid. Although this addition of H_2_SO_4_ also increased the ionic strength, previous studies found that Si(OH)_4_ polymerization in the presence of H_2_SO_4_ is minimal^36^. The present results (Fig. 3) show a moderate stabilizing effect of H_2_SO_4_ on dissolved Si, based on a 3.5% difference between the sensitivities of Si calibrations in H_2_SO_4_ or BgS.

The higher the excess KOH concentrations, the lower the Si recovery was, which is in line with the abovementioned destabilization of Y and stabilization of Si in high KOH environments. The Si recovery dropped by 15% in samples containing 0.1 M KOH compared to 1.0 M KOH. Hence, it is important to add the same concentration of KOH to the calibration in case the samples require KOH concentrations >0.1 M for digestion to account for this matrix effect. Finally, the SiO_2_ polymerization is accelerated by high Si(OH)_4_ concentrations^36^. We found plasma instability starting from 4000 µg Si L^−1^ upwards, and therefore limited routine concentrations to <1000 µg Si L^−1^.

#### Sample storage

**Figure 5:**
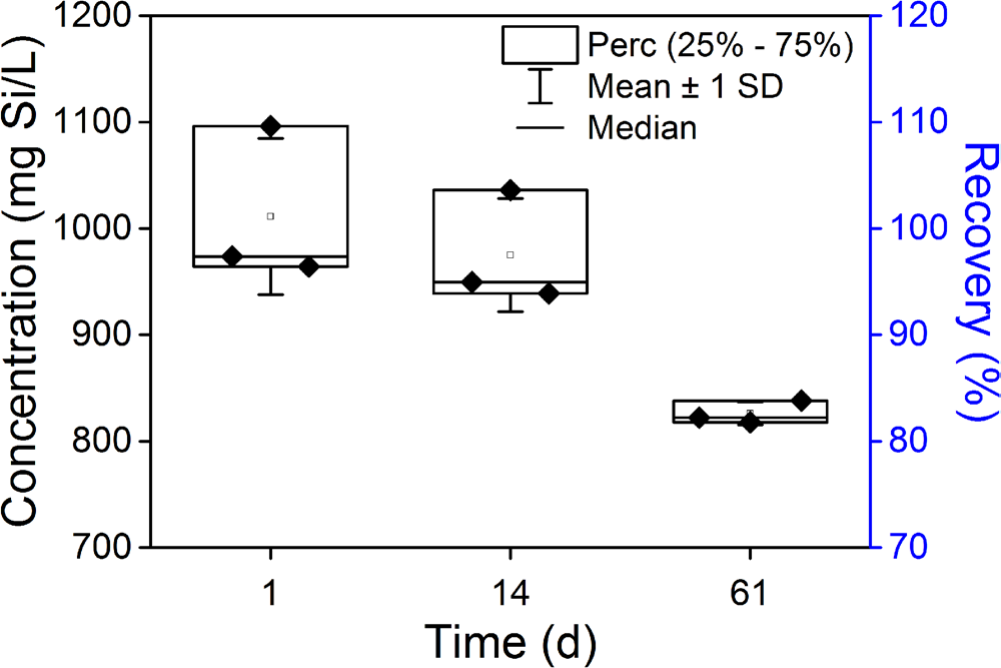
Stability of digestates containing hydrolytically degraded SiO_2_ over time. The concentration is proportional to the recovery: the data can be read from both y-axes. Certified Si standard solutions digested according to method KOH0.1 (Table 1) and stored at room temperature were measured at different time points after digestion. The storage time significantly affected the concentration after sixty-one days, but not after fourteen days (analysis of variance, p < 0.02, Tukey’s post-hoc test, p > 0.69).

Storing samples for extended periods showed that digestates could be analyzed after up to two weeks without a statistically significant loss of recovery. A 3.6% decrease of Si recovery from 101.1% to 97.5% was observed between day 1 and at day 14 (Fig. 5, analysis of variance (ANOVA) and Tukey’s *post-hoc* test, p > 0.69). The recovery after 61 days of storage showed a significantly (18.3%, p < 0.031) reduced Si recovery of 82.6%. Such extended periods of storage of concentrated digestates at room temperature (RT) significantly affect the freely dissolved Si in the sample (ANOVA p < 0.02), likely due to re-polymerization. We noted improved stability of (1) refrigerated, (2) diluted, (3) low ionic strength, and (4) low pH samples. All these three conditions are known to push the equilibrium of polymerized SiO_2_ towards Si(OH)_4_^36^.

### Method applicability

#### Suspensions containing fumed SiO_2_-NPs (Aerosil^®^)

The recoveries for fumed SiO_2_-NP suspensions digested using the method KOH1.0 (Table 1) are summarized in Table 2. The fumed SiO_2_-NP stock suspensions mainly contained aggregates (hydrodynamic diameter 267 nm) of smaller primary NPs 13 ± 5 nm in diameter (Supplementary Fig. S1 and Supplementary Table S1). We chose a harsher KOH concentration of 1.0 M for fumed SiO_2_-NPs due to the expected poorer solubility of the non-porous and less hydroxylated fumed SiO_2_-NPs compared to the more porous and more hydroxylated colloidal SiO_2_-NPs^8,32,37^. While the specific surface area is, for the present particle sizes and fractal dimensions, expected to be higher for the fumed SiO_2_-NPs (200 m^2^ g^−1^) than the colloidal SiO_2_-NPs (~23–32 m^2^ g^−1^ based on literature for colloidal particles of smaller size)^32^, both the lower surface hydroxylation and lower porosity of fumed SiO_2_-NPs can hamper the base-catalyzed hydrolytic degradation due to the postulated mechanism of amorphous SiO_2_ dissolution that first requires hydration and hydrolysis of amorphous siloxane networks into silanols before the nucleophilic attack of OH^−^ ^31^. Also, suspensions of pre-digested, oven-dried SiO_2_-NPs formed acidic suspensions, which partially neutralized the added KOH in initial attempts to use 0.1 M KOH for digestion. Using 1.0 M KOH, we obtained a recovery of 114 ± 25% for fumed SiO_2_-NPs, and the same digestion at RT without microwave 105 ± 1.4% (Table 2). This elevated recovery (not significantly higher than 100%, one sample T-test, p > 0.22) may be a result of slightly less stabilized free Si ions than Y ions in the digestates, which were slightly more acidic than the calibrations. In samples digested using KOH concentrations ≤0.5 M, recoveries remained <85% in ICP-OES measurements (Table 2), confirming that significant matrix effects occur due to excess KOH, as discussed in section *Repeatability and Recovery*, only in SiO_2_ samples that are digested in >0.1 M KOH.

#### SiO_2_ in complex matrices

The performance of the method in SiO_2_-containing complex matrices tested is shown in Table 2 (cell culture medium, tomato sauce, potato seasoning). We observed low recoveries for the digestion of SiO_2_ in complex samples using 0.1 M KOH in preliminary tests. By using the method KOH0.5 on the SiO_2_-NP-spiked cell culture medium samples, we obtained a recovery of 84 ± 20% of SiO_2_ without pre-digestion (Table 2). The large statistical scatter can be connected to the complex formulation of the cell culture media Dulbecco Modified Eagle Medium (DMEM). Among many amino acids and vitamins, DMEM also contains ~10 g L^−1^ of dissolved inorganic salts, of which 3.6 g L^−1^ is sodium, which is notorious for causing high variability in ICP-OES measurements^30,38^. The present results show that the KOH digestion of SiO_2_-NPs in a serum-free cell culture medium delivered, despite some variability, an acceptable accuracy and recovery.

For the food matrix samples, *i*.*e*. the tomato sauce spiked with colloidal SiO_2_-NPs and the potato seasoning, the matrix was first digested in HNO_3_ to isolate the SiO_2_-NPs (*i*.*e*. pre-digestion) and then these NPs were dissolved by KOH (refer to *Experimental Section*). As with DMEM, we had to use higher KOH concentrations of 1.0 M to get satisfactory recoveries. We obtained a recovery of 124 ± 5% and 95 ± 13% for colloidal SiO_2_-NPs in tomato sauce and food grade SiO_2_ (E551) in potato seasoning, respectively. The recoveries of both samples (tomato sauce, potato seasoning) were calculated relative to the mass of remaining solids after the first acid-mediated digestion step, as SiO_2_ was the sole remainder detected by energy-dispersive X-ray spectroscopy (EDX) after the harsh HNO_3_ pre-digestion (data not shown). According to the literature, the natural Si concentration in tomatoes is maximally ~61 mg kg^−1^ ^39^, corresponding to ~31 µg natural Si in the analyzed mass of tomato sauce. The high recovery of 124 ± 5% for colloidal SiO_2_-NPs spiked into the tomato sauce (Table 2) indicates that additional natural SiO_2_ was detected in the tomato sauce. The recovery of 95 ± 13% SiO_2_ found for the potato seasoning (Table 2) corresponds to a total of 4.8 g SiO_2_ kg^−1^ for the potato seasoning. Sodium residues from the pre-digestion can be the reason for the more variable results compared to the other tested matrices, in line with the results for DMEM, and as also reported by Frantz *et al*.^30^. The quantity of the anti-caking agent was not indicated on the potato seasoning package. However, our results are in good agreement with Si analyses of related products in the literature^40^.

#### SiO_2_-NP digestion at room temperature—ICP-OES (HF-free) vs. colorimetry (not HF-free)

For colloidal SiO_2_-NPs in a simple matrix, the microwave digestion is replaceable by an RT digestion overnight in 0.1 M or 1.0 M KOH, without much reduction in recoveries (Table 2). Colloidal SiO_2_-NPs digested in 1.0 M KOH at RT yielded a recovery of 84 ± 5% compared to 85 ± 2% for 0.1 M KOH in the microwave (both measured by ICP-OES). This demonstrates that porous, almost entirely hydroxylated colloidal SiO_2_-NPs are digestible at RT without expensive instrumentation, and confirms reports by Tanakaa and co-workers, who found that silica gel dissolves in 0.1 M KOH without the aid of microwave irradiation^18^.

The efficiency of ICP-OES and colorimetry in detecting SiO_2_-NPs was directly compared for samples digested using the method RT + KOH1.0 (Tables 1 and 2). Using colorimetry, the recovery for fumed SiO_2_-NPs was lower (76 ± 9%) than for ICP-OES (105 ± 1.4%). Also, for the fumed SiO_2_-NPs, the recovery was only 67 ± 2% when digested in 0.1 M KOH at RT (Table 2), revealing a limitation of the digestion methods at RT for fumed SiO_2_-NPs and colorimetry that only detects fully dissolved orthosilicic acid or small Si oligomers^21^. Despite the larger specific surface area, the non-porous, less hydroxylated fumed SiO_2_-NPs were, in agreement with Zhang and co-workers^37^, harder to completely digest and required the harsher 1.0 M KOH conditions, in contrast to the more soluble porous, more hydroxylated colloidal SiO_2_-NPs. The ICP-OES method was more robust in detecting incompletely digested SiO_2_ at RT: a high recovery was found for fumed SiO_2_-NPs of 105 ± 1.4% in 1.0 M KOH.

The trend in the recovery of the two detection methods for colloidal SiO_2_-NPs was inverse: despite milder digestion conditions (0.1 M KOH), colorimetry detected more Si (111 ± 7%) than ICP-OES (84 ± 5%, 1.0 M KOH). The simplest explanation for this seemingly contradictory result is that the harsh 1.0 M KOH conditions readily dissolved the colloidal SiO_2_-NPs, and because the easier to dissolve colloidal SiO_2_-NPs did not consume all of the 1.0 M KOH, the excess KOH negatively affected the ICP-OES recovery. This confirms the earlier finding that, for colloidal SiO_2_-NPs, KOH concentrations <0.5 M are sufficient for ICP-OES analysis and excess KOH should be avoided.

The present results show that the ICP-OES detection of Si is more widely applicable than colorimetry because, despite satisfactory recoveries, the quantification *via* colorimetric detection of Si has several limitations. First, as mentioned before, the colorimetric quantification of Si suffers from a wide variety of interferences^20,41^ and exclusively detects fully dissolved Si(OH)_4_ or small oligomers^21^. Second, the present colorimetric determination of Si employed a four-fold higher dilution factor (105) compared to ICP sample preparation (25). Based on the LODs in Table 2, this results in an estimated detectable concentration for the colorimetry of >15–32 mg SiO_2_ L^−1^, and for the ICP-OES of >1.7–7.4 mg SiO_2_ L^−1^, depending on the sample matrix. The high detection limit for the colorimetry makes it challenging to detect Si in samples with low SiO_2_ concentrations of <15 mg SiO_2_ L^−1^ without additional pre-concentration steps as used *e*.*g*. by Rimmelin-Maury and co-workers^6^. Future development of the KOH digestion method for colorimetry should, therefore, focus on reducing the LOD by reducing this dilution factor or including pre-concentration steps. Finally, the digestion protocol for colorimetry uses ammonium fluoride at a low pH, which raises concerns of *in situ* hydrofluoric acid formation due to its pK_a_ of ~3.17.

## Conclusion

**Figure 6:**
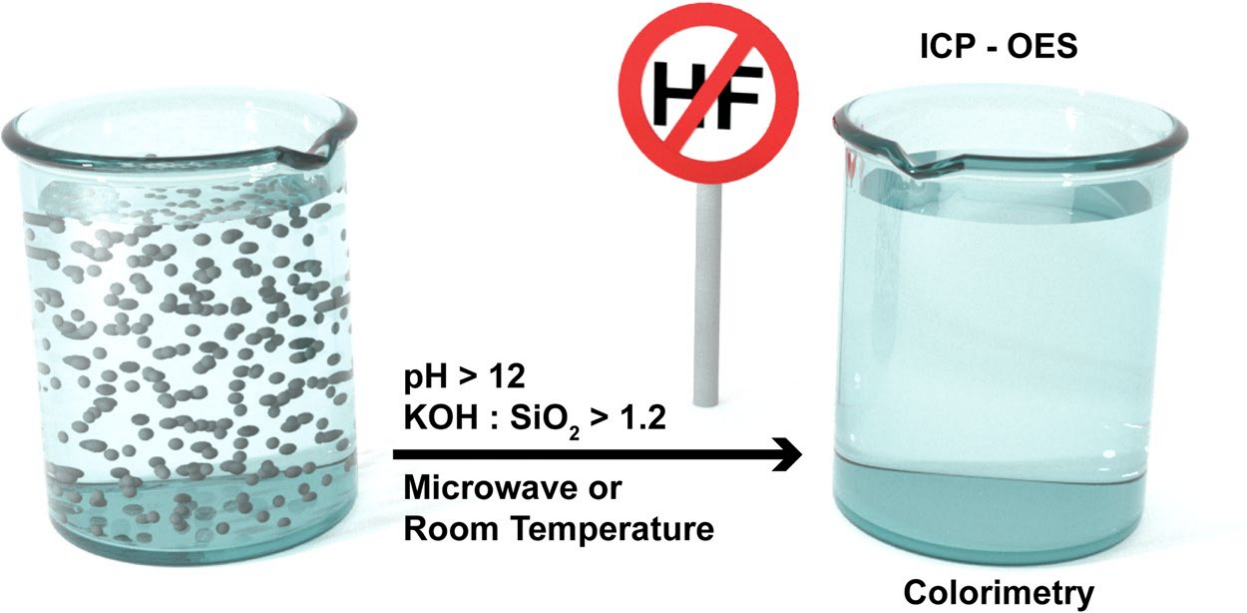
Graphical abstract showing the main findings of this study. A hydrofluoric-acid-free digestion method to quantify SiO_2_ nanoparticles in different matrices was developed for optical emission spectrometry and cross-validated by colorimetry and an HF approach.

Herein, we report a series of methods using basic KOH digestion to quantify Si in a broad variety of samples. Digested samples containing particulate amorphous SiO_2_ or Si(OH)_4_ could be quantified by ICP-OES or colorimetry (Fig. 6). The method was successfully applied in samples of low and high complexity including aqueous colloidal or fumed SiO_2_-NP suspensions, SiO_2_-NP-spiked cell culture media, SiO_2_-NP-spiked tomato sauce, and potato seasoning containing food grade SiO_2_ (E551). SiO_2_ dissolved at a minimum KOH: SiO_2_ ratio of 1.2 at pH values >12. The complexity of the sample matrix and the manufacturing process of the SiO_2_ under investigation both affect the Si recovery. Recovery can be improved by controlling the excess of KOH. The different optimal KOH concentrations reflect trade-offs between high excess KOH and harsh pH conditions that favor the rapid dissolution of less porous and less hydroxylated fumed SiO_2_-NPs and Si in more complex matrices; and low excess KOH concentrations, where less matrix effects occur. In case KOH concentrations >0.1 M are used, the calibration has to be prepared in the same concentration of KOH to account for these matrix effects (matrix-matched calibration). Some limitations of the method to be addressed in follow-up studies are the efficiency for larger SiO_2_ particles ≥397 ± 22 nm, long term sample storage, the applicability of the method in sera (*e*.*g*. 10% fetal calf or bovine serum), and the differentiation of dissolved and particulate SiO_2_ species that can be addressed by size fractionation steps prior to further analysis.

Both detection by ICP-OES or colorimetry yielded satisfactory recoveries of up to 100% for SiO_2_-NPs ≤397 ± 22 nm. This shows that our approach without HF can lead to recoveries and detection limits comparable to the state-of-the-art colorimetry method involving HF that was tested here to validate our method^42^. While colorimetry is easy and fast for simple matrices and colloidal SiO_2_-NPs and also feasible with a preceding HF-free KOH digestion, the ICP-OES method presented here is completely hydrofluoric acid-free, independent from color interferences due to matrix components such as Fe, nitrates, and sugars, and more accurate than colorimetry for incompletely digested nanoparticles (*e*.*g*. from fumed SiO_2_). Thus, the hydrofluoric acid-free SiO_2_ dissolution and quantification methods presented here are simple to implement alternatives to current standard procedures and applicable in fields such as biomedical sciences and environmental chemistry where SiO_2_-NP quantification in complex matrices is important.

## Method Section

### Materials, chemicals, and matrices

Commercially available fumed (pyrolytic) SiO_2_-NPs (Aerosil^®^ 200, 98% SiO_2_, specific surface area of 200 m^2^ g^−1^) were purchased from Evonik (former Degussa). Fumed SiO_2_-NPs are produced by continuous flame hydrolysis, are reported to be non-porous by the manufacturer and Mebert and co-workers^8^, and are less hydroxylated than colloidal SiO_2_-NPs^37^. All chemicals used were *per analysis* grade unless it is stated otherwise. Water was pre-purified by a Milli-Q system (18.2 MΩ.cm arium 611DI, Sartorius Stedim Biotech, Germany). Dialysis membranes were purchased from Roth (Membra-Cel™, 14 kDa cut-off).

Both cell culture medium and food matrices are relevant chemically complex matrices that reportedly pose significant analytical challenges for NP analytics^43,44^. We selected three representative complex matrices according to the following criteria: (1) the cell culture media DMEM is widely used in *in vitro* NP-cell interaction studies^45^; (2) tomato sauce is a typical food matrix containing with <61 mg kg^−1^ comparatively little SiO_2_^39^; and (3) potato seasoning is a foodstuff where E551, *i*.*e*. food grade SiO_2_, was listed on the packaging as an anti-caking ingredient. The potato seasoning (Qualité & Prix Country Potato Seasoning Blend, Germany) and the tomato sauce (Cirio Rustic Tomato Purée, Italy) were purchased from a local supermarket.

### Colloidal SiO_2_-NP synthesis

Colloidal SiO_2_-NPs were synthesized *via* a co-condensation reaction adapted from Stöber *et al*.^46^. Briefly, ethanol (522 mL, absolute, Honeywell), ammonia (122.7 mL, 1.65 mol, 25% aqueous solution, Merck), and water (40.5 mL, MilliQ) were mixed and heated to 60 °C. The mixture was stirred at that temperature for 1 h to equilibrate. Tetraethyl orthosilicate (67.5 mL, 302 mmol, Sigma-Aldrich) was added, and the mixture was stirred at 60 °C overnight. The mixture was allowed to cool to RT, and the NPs were washed three times by centrifugation (Thermo Scientific, F15-8 × 50cy fixed-angle rotor, 5000 × *g*, 10 min) and redispersed in water. The final opaque SiO_2_-NP suspension (500 mL) contained 23.1 g SiO_2_ kg^−1^, as determined gravimetrically by drying aliquot volumes of the suspension. Due to the sol-gel manufacturing process, colloidal Stöber SiO_2_-NPs are more porous and almost fully hydroxylated compared to the fumed SiO_2_-NPs^8,32,37^.

### Nanoparticle characterization

The SiO_2_-NPs were characterized by TEM (primary particle diameter), and dynamic light scattering (DLS, hydrodynamic particle diameter, surface charge). The results are summarized in Supplementary Fig. S1 and Supplementary Table S1. For TEM analysis, samples were prepared by diluting NP suspension (1 μL) with ethanol (5 μL, absolute, Honeywell) for SiO_2_-NPs and water for fumed SiO_2_-NPs directly on the TEM grids (carbon film, 300 mesh on Cu, Electron Microscopy Sciences) and wicking remaining liquid using a precision wipe tissue (Kimtech Science). The TEM images were recorded in 2048 × 2048 pixel resolution (Veleta CCD camera, Olympus) on a FEI Tecnai Spirit TEM, operating at an acceleration voltage of 120 kV. The DLS samples were diluted with water (1% v/v) and measured on a Brookhaven Particle Size Analyzer Plus90 (USA) (scattering angle 90°, 1 min acquisition, 10 repetitions). The size distribution of the particles was analyzed by computer-assisted particle size analysis software (imageJ, plugin: psa-r12)^47^, applied to the TEM micrographs.

### Digestion pretests to find the KOH concentration for complete SiO_2_ dissolution

A series of digestion methods (throughout the text referred to as High_SiO_2_) was tested to find the highest SiO_2_ mass and lowest KOH concentration that allowed for complete solubilization of all SiO_2_ nanoparticles in the sample. Colloidal SiO_2_-NP suspensions (433–2165 µL of a 23.1 g SiO_2_ kg^−1^ suspension, equivalent to 10, 20, 30, and 50 mg of SiO_2_) were weighed into the PTFE microwave vessels, and KOH (10 mL, 0.05, 0.1, 0.5, or 1.0 M) was added (Table 1). The mixtures were sealed and digested in the microwave (details below). The digestates were measured by DLS (particle counts per second) and visualized using TEM to detect undigested SiO_2_-NPs.

### Digestion methods investigated for ICP-OES

Figure 1 shows the key steps, and Table 1 the reagents and concentrations used in the different digestion protocols investigated. All microwave digestions were conducted using an Anton Paar Multiwave PRO, equipped with a 24HVT50 rotor holding 25 mL PTFE microwave vessels with pressure-activated-venting caps (PTFE-TFM, max. pressure 40 bar). All microwave runs consisted of a temperature ramp to 200 °C for 7 min followed by a temperature hold for 7 min and concluded by a cooling segment until the internal temperature in all containers reached 70 °C (Supplementary Fig. S2) resulting in a total duration of the microwave digestion of ~28 min. The power limit for all runs was set to 1500 W. If not stated otherwise, digested samples and calibrations were stored at RT and analyzed by ICP-OES within 24 h. Digestates spiked with internal Y standard and stabilized in acidic BgS and were stored in the fridge. The background equivalent concentrations (BEC), the limits of the detection (LOD) and limits of quantification (LOQ) were calculated by adding three times the BEC standard deviation to the BEC (LOD), and ten times the BEC standard deviation to the BEC (LOQ).

#### KOH0.1-KOH1.0

These methods served to assess the Si recovery for (a) 120 colloidal SiO_2_-NP suspensions in the range of 1.88 to 8.53 mg L^−1^ SiO_2_ in the course of ten experiments (method KOH0.1); (b) a different SiO_2_ source (fumed SiO_2_-NPs, method KOH0.1 and KOH1.0); (c) more complex matrices spiked with colloidal SiO_2_-NPs in the concentration range of 0.4–1.7 mg L^−1^ SiO_2_ (0.5 g of tomato sauce and 0.5 mL of cell culture medium, pre-digestion except for cell culture medium as explained below, then methods KOH0.1-KOH1.0); and (d) the pre-digested commercial potato seasoning (0.5 g) with an unknown Si concentration treated equally to the complex matrices in (c). For all methods, SiO_2_-NP stock suspensions (25 mg SiO_2_ kg^−1^) were weighed into the PTFE microwave vessels and diluted with water to ~2 g. Aqueous KOH (3 mL, 0.1, 0.5, or 1.0 M, respectively, Table 1) was added, and the mixtures were prepared for the microwave run. The digestates were transferred to conical tubes (polypropylene, Falcon^®^) and acidified by H_2_SO_4_ (2.25 M) to pH 1–2. Internal standard (yttrium, 50 mg L^−1^ solution in 2% HNO_3_/0.5% HCl, 100 μL) was spiked, and the samples were topped off with water to 10 mL for the ICP-OES analysis.

#### RT + KOH0.1, RT + KOH1.0

Here, the SiO_2_ digestion at RT was tested, and the Si detection of ICP-OES was compared with colorimetry. For the ICP measurements, stock suspensions (25 mg SiO_2_ kg^−1^ in water) of either colloidal or fumed SiO_2_-NPs were mixed with KOH (3.0 mL, 1.0 M) and stirred overnight at RT (Table 1). Then, H_2_SO_4_ (2.25 M) was added until pH 1–2 was reached. Internal standard (yttrium, 50 mg L^−1^ solution in 2% HNO_3_/0.5% HCl, 100 μL) was spiked, and the resulting digestates were topped off with water to 10 mL for the ICP-OES analysis.

#### Pre-digestion of samples containing food matrix

The colloidal SiO_2_-NP-spiked tomato sauce samples and the food grade SiO_2_-NP containing potato seasoning were pre-digested according to a procedure for food analysis established in our laboratory. In pretests, we found that for these food matrices, the method KOH0.1 to KOH1.0 was not suitable due to the high solid content. We therefore used a two-step digestion for these samples, consisting of an acidic pre-digestion of the food matrix followed by KOH digestion of the oven-dried SiO_2_-containing residue. Briefly, for the pre-digestion, the sample (~0.5 g) was added to the PTFE microwave vessels and nitric acid (63%, 3 mL) was added. The closed vessels were heated in the microwave (700 W, 10 min at 60 °C) without previously running a ramp. After this run, the vessels were opened to release nitric oxide gases, closed again, and heated in the microwave (800 W) according to the following program: ramp (90 °C, 5 min), hold (2 min), ramp (180 °C, 6 min), hold (15 min) and cool to 70 °C. The cooled digestates were transferred into 15 mL conical tubes (polypropylene, Falcon^®^) and diluted with water to 5 mL. The digested samples were cleaned by centrifugation at 8000 × *g* for 10 min at 4 °C and redispersed in 1 mL of water. The centrifugation-redispersion cycle was repeated until the pH of the suspensions reached 5–6. The water was evaporated in an oven and the resulting Si-containing solids were operationally defined to consist of 100% SiO_2_, as an energy dispersive X-ray spectrometric (EDX) elemental analysis found no impurities. These solids were used to prepare stock suspensions in water for quantification experiments using the basic digestion methods KOH1.0-KOH0.1 and subsequent ICP-OES analysis.

### ICP-OES Si Quantification and Calibrations

The ICP-OES analyses of Si were performed on a Perkin Elmer Avio-200 (radio frequency power 1500 W, gas flow rates 8 L/min (Ar, plasma), 0.2 L/min (N_2_, auxiliary), pump 0.8 L/min (nebulizer), concentric glass nebulizer, cyclonic glass spray chamber, spectral lines: Si 251.611 nm, Y 371.029 nm). Certified reference element standards (TraceCERT^®^; Y, 989 mg kg^−1^ in 2% HNO_3_; Si, 975 mg kg^−1^ in 2% NaOH, Sigma-Aldrich) were diluted gravimetrically in an acidic BgS to a concentration of 50 mg kg^−1^.

Four types of Si calibrations with increasing complexity were prepared using the same volumes and concentrations as in the digestion method KOH0.1 to assess the effects on the Si sensitivity of the ICP-OES for samples in different acids, in KOH matrix, and digested in the microwave. The four Si calibrations were Si in water and H_2_SO_4_ (short: water + H_2_SO_4_); Si in BgS; Si in water and KOH (3 mL, 0.1 M), acidified by H_2_SO_4_ (short: matrix-matched + H_2_SO_4_); and Si in water and KOH (3 mL, 0.1 M) digested in the microwave, and acidified by H_2_SO_4_ (short: matrix-matched + H_2_SO_4_ + digested). The background was accounted for by subtraction of the blank concentration.

### Sample preparation for colorimetric SiO_2_ analysis

To test the suitability of the KOH digestion method for colorimetry, and to cross-validate the ICP-OES results using a conventional approach involving hydrofluoric acid, we quantified the dissolved silicon dioxide according to a modified version of the colorimetric method based on the blue molybdosilicic acid complex (Fig. 1)^19^. For the digestion, lyophilized colloidal SiO_2_-NPs (2.0 mg, 33.0 µmol SiO_2_) or fumed SiO_2_-NPs (1.7 mg, 28.3 µmol SiO_2_) were suspended in 0.1 M KOH (20 mL) for the colloidal SiO_2_-NPs or 1.0 M KOH (20 mL) for the fumed SiO_2_-NPs and stirred overnight at RT. All the resulting digestates were then diluted to a final concentration of 0.1 M KOH. From here, we followed the colorimetric SiO_2_ analysis protocol reported by Yang *et al*.^19^ using 5 mL of the colloidal SiO_2_-NP digestate and 9 mL of the fumed SiO_2_-NP digestate (concentration: 9.2–92 colloidal SiO_2_ L^−1^, and 9.2–14.4 mg fumed SiO_2_ L^−1^, respectively). Water (5 mL), HCl (1 M, 5 mL) and NH_4_F (1 M, 1 mL) were added, and the mixtures were stirred at 25 °C in a water bath for 45 min. Mixing a 5-fold excess of HCl with NH_4_F produces HF *in situ* due to the pK_a_ of HF of 3.2 and the much stronger pK_a_ of −6.3 of HCl. Boric acid (0.5 M, 10 mL) was added, and the mixture was stirred for 30 min. The pH was adjusted to 1.4 with NaOH (1 M). EtOH (5 mL) and ammonium molybdate (5% (w/v), 7.5 mL) were added, and the mixture was continued to stir for 20 min. A combination of oxalic and tartaric acid (1:1, 0.5 M, 5 mL) and ascorbic acid (2 w/v%, 2.5 mL) was added to minimize false-positives due to phosphate^48,49^. The mixture was stirred for 20 min and measured at 812 nm wavelength in a quartz cuvette (Hellma Analytics, Germany) by UV-Vis (V-670, Jasco, USA).

### Sample storage tests

While it is preferable to measure freshly prepared samples, knowing the maximum amount of time a sample can be stored without a significant decrease in Si recovery can help to simplify the planning of laboratory work. We therefore investigated the stability of digestates prepared according to method KOH0.1 over an extended period (2 months). The digestates were stored at RT (21 ± 1 °C) in 50 mL polypropylene conical tubes (Falcon^®^, Corning, NY, USA) and measured 1, 14, and 61 days after the digestion. The samples contained Si reference standard solution at 35.6 mmol L^−1^ (TraceCERT^®^, 1000 ± 2 mg L^−1^ Si in NaOH, Sigma-Aldrich, Switzerland). For the analysis by ICP-OES, the digestates were diluted in an acidic BgS to an expected final Si concentration that fell into the range of the Si calibration (Fig. 5). These diluted digestates were spiked with 500 µg L^−1^ Y and analyzed within 24 h by ICP-OES.

## Supplementary information

Supplementary Information

## Data Availability

The data generated or analyzed during this study are included in this article and its Supplementary Information Files. Raw data sets analyzed during this study are available from the corresponding author upon reasonable request.
