# Supplementary material for: A hydrofluoric acid-free method to dissolve and quantify silica nanoparticles in aqueous and solid matrices
Source: Sci Rep. 2019 May 28;9:7938. doi: 10.1038/s41598-019-44128-z (PMC6538650; doi:10.1038/s41598-019-44128-z)
Supplement: Supplementary file 1 — Supplementary Information [file 41598_2019_44128_MOESM1_ESM.pdf]

A hydrofluoric acid-free method to dissolve and quantify silica nanoparticles in aqueous and solid matrices

David Bossert,[1] Dominic A. Urban,[1] Mattia Maceroni,[1] Liliane Ackermann-Hirschi,[1] Laetitia Haeni,[1] Phattadon Yajan,[1] Miguel Spuch-Calvar,[1] Barbara Rothen-Rutishauser,[1] Laura Rodriguez-Lorenzo,[1] Alke Petri-Fink, *[1,2] Fabienne Schwab*[1]

Affiliations:

[1]Adolphe Merkle Institute, University of Fribourg, Chemin des Verdiers 4, 1700 Fribourg, Switzerland

[2]Chemistry Department, University of Fribourg, Chemin du Musée 9, 1700 Fribourg, Switzerland

*Correspondence: alke.fink@unifr.ch +41 26 300 9501, fabienne.schwab@unifr.ch +41 26 300 9510

SUPPLEMENTAL INFORMATION

This supplemental information contains more details on the nanomaterial characterization (**Supplementary Figure S1 + Table S1**), the temperature and pressure profile of the microwave digestion (**Supplementary Figure S2**), and data on the calibration parameters (**Supplementary Table S2**).

Supporting Information

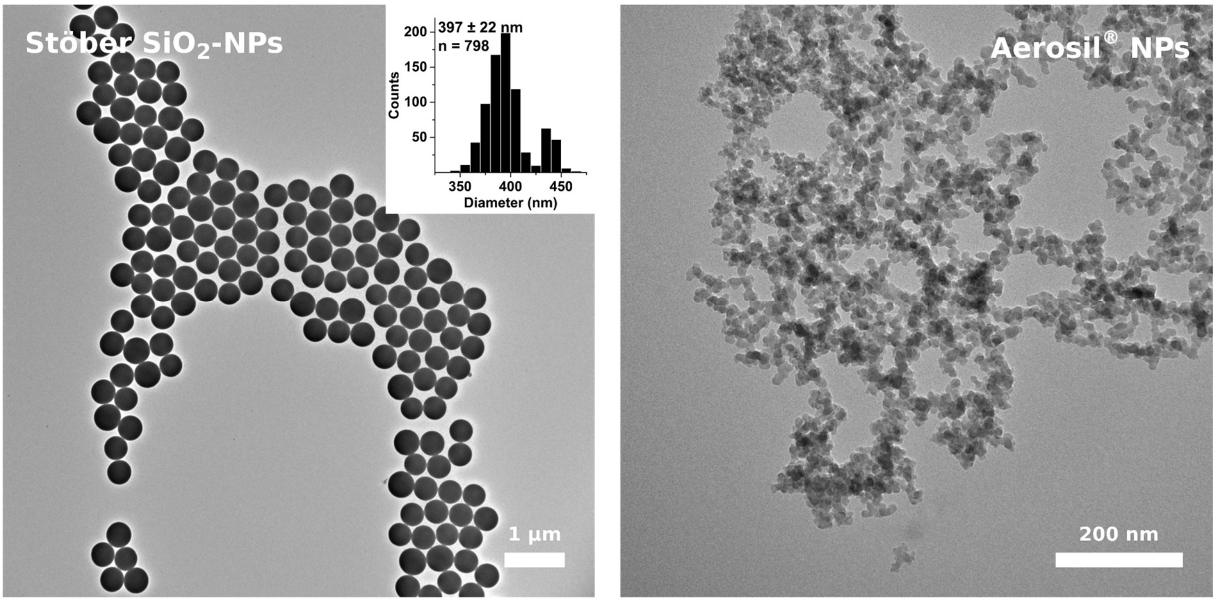

Supplementary Figure S1: Transmission electron microscopy (TEM) characterization of in house synthesized amorphous colloidal silica nanoparticles (SiO_2-NPs) and commercially available amorphous fumed Aerosil® 200 SiO_2-NPs (Evonik, former Degussa).

Supplementary Table S1: Overview of particle parameters as determined by transmission electron microscopy (TEM) and dynamic light scattering (DLS) of the amorphous silica nanomaterials. PDI: Polydispersity index. Zeta: Zeta potential, which represents the surface charge of the particles.

	d_{TEM} (nm)	d_{DLS} (nm)	PDI	Zeta (mV)
Colloidal SiO_2-NPs	397 ± 22	412	0.005	-82.7 ± 7.7
Fumed SiO_2-NPs	13 ± 5[a]	267	0.22	-42.4 ± 2.8

[a]diameter of primary particles.

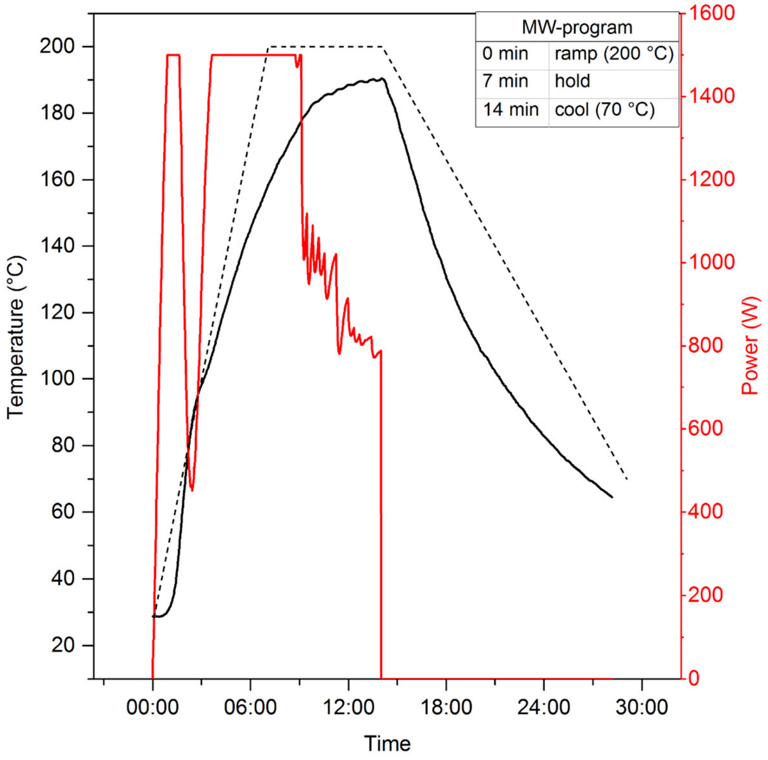

Supplementary Figure S2: Temperature and power measured in the microwave PTFE digestion vessels. The temperature in each vessel was monitored by a built-in infrared sensor. The graph shows the temperature in black and the applied power in red for the 24 PTFE microwave vessels in one run. The dotted line represents the programmed route.

Supplementary Table S 2: Fitting parameters for the different types of calibrations measured by inductively coupled plasma – optical emission spectrometry (ICP-OES) and colorimetry.

	Si												Y						
	Sensitivity (a.u. µg^{-1} L)			Intercept (a.u.)			R^2 (-)	Blanks (µg L^{-1})					Sensitivity (a.u. µg^{-1} L)			Intercept (a.u.)			R^2 (-)
**ICP-OES**	mean	±	s.d.	mean	±	s.d.		BEC	±	s.d.	LOD	LOQ	mean	±	s.d.	mean	±	s.d.	
matrix-matched + H_2SO_4 + digested	652	±	9.5	8731	±	5342	0.9987	23.2	±	1.3	27	35.8	17279	±	368	-414464	±	208303	0.9977
matrix-matched+H_2SO_4	619	±	4.7	10267	±	2633	0.9997	18.4	±	0.8	20.7	26.2	16467	±	211	-52758	±	119408	0.9992
background solution	602	±	3.4	14898	±	1898	0.9998	26.1	±	0.2	26.6	27.8	18204	±	321	-115106	±	181764	0.9984
water+H_2SO_4	624	±	3.6	13501	±	2031	0.9998	15.9	±	0.1	16.3	17.4	17692	±	170	18118	±	95968	0.9995
	Sensitivity (a.u. mg^{-1} L)			Intercept (a.u.)															
**Colorimetry**	mean	±	s.d.	mean	±	s.d.													
RT+KOH0.1	0.722	±	0.03	-0.0394	±	0.076	0.9960	53.6	±	7.8	69.2	123							
RT+KOH1.0	0.715	±	0.02	-0.0459	±	0.037	0.9980	56	±	9	82	143							

a.u.: arbitrary units. s.d.: standard deviation. BEC: background equivalent concentration. LOD: instrument limit of detection. LOQ: instrument limit of quantification. RT: room temperature.
